# Backward Secondary-Wave Coherence Errors in Photonic Bandgap Fiber Optic Gyroscopes

**DOI:** 10.3390/s16060851

**Published:** 2016-06-08

**Authors:** Xiaobin Xu, Ningfang Song, Zuchen Zhang, Jing Jin

**Affiliations:** Department of Opto-electronics Engineering, Beihang University, Beijing 100191, China; songnf@buaa.edu.cn (N.S.); zzc1022@163.com (Z.Z.); jinjing@buaa.edu.cn (J.J.)

**Keywords:** photonic bandgap fiber, fiber optic gyroscope, bias error

## Abstract

Photonic bandgap fiber optic gyroscope (PBFOG) is a novel fiber optic gyroscope (FOG) with excellent environment adaptability performance compared to a conventional FOG. In this work we find and investigate the backward secondary-wave coherence (BSC) error, which is a bias error unique to the PBFOG and caused by the interference between back-reflection-induced and backscatter-induced secondary waves. Our theoretical and experimental results show a maximum BSC error of ~4.7°/h for a 300-m PBF coil with a diameter of 10 cm. The BSC error is an important error source contributing to bias instability in the PBFOG and has to be addressed before practical applications of the PBFOG can be implemented.

## 1. Introduction

Current fiber optic gyroscopes (FOGs) have very good performance [1], but inevitably they still have large Shupe effects, Faraday effects and radiation effects due to the fact that conventional silica fiber is very sensitive to these environments. Photonic bandgap fibers (PBFs), a novel kind of fiber in which the light travels not in a conventional silica core but in an air core [2,3,4], have attracted considerable interest owing to their unique optical properties such as significantly lower nonlinear coefficients and especially, their improved adaptability to temperature, magnetic fields and radiation in comparison with the properties of silica fibers [5,6].

**Figure 1:**
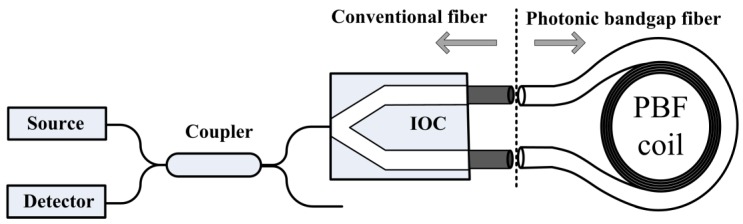
Diagram of a photonic bandgap fiber optic gyroscope.

A photonic bandgap fiber optic gyroscope (PBFOG), as illustrated in Figure 1, consists of a broad-spectrum source, a coupler, an integrated optic chip (IOC), and a PBF coil [7,8]. A previous study [9] has reported reductions in the errors induced by the Kerr effect (>170), Shupe effect (~6.5), and Faraday effect (>20) in a PBFOG when compared with the corresponding error values of a conventional FOG. Therefore, the PBFOG represents a substantially improvement of these key properties compared to a FOG, and it has excellent prospects, but as a new kind of FOG, it definitely exhibits some different errors and noises from those which are normally observed in a traditional FOG owing to the use of the PBF. References [7,8] have investigated shot noise, backscattering noise which is defined as the error due to coherence interference between the backscattered waves and primary waves and which also exists in resonant fiber optic gyroscopes using an air-core fiber [10,11], electronic noise, and reflection-induced intensity noise; further, the polarization non-reciprocity error has also been investigated [12]. In this study, for the first time to the best of our knowledge, we find and investigate the backward secondary-wave coherence (BSC) error, which is defined as a bias error caused in the PBFOG by interference between back-reflection-induced and backscatter-induced secondary waves.

## 2. Theoretical Analysis

In the PBFOG, the pigtails of the IOC are conventional fibers that have a Ge-doped SiO_2_ core, but the coil comprises a commercially available 7-cell air-core PBF with hexagonal air holes triangularly arranged in the cladding [12]. According to the measurement results in our previous study [13], a strong reflection inevitably occurs at the two fusion splicing points between the PBF coil and the IOC pigtails. Both back-reflection-induced secondary waves have very large magnitudes (only about 3–10 times smaller than that of primary waves for a 300-m coil composed of a PBF with a loss of 20 dB/km). Obviously, those back-reflection-induced secondary waves produce an offset at the detector and add intensity-type noise because the coherence length of the broad-spectrum source is always considerably less than the path mismatch length between the two IOC pigtails, and thus, there is no interference between these two sets of secondary waves [7,9]. However, we found that the strong back-reflection-induced secondary waves at one fusion splicing point inevitably interfere with the backscatter-induced secondary waves within the other pigtail, thereby causing a BSC error. This BSC error is a phase-type error that does not exist in a conventional FOG, and it seriously affects the PBFOG performance.

**Figure 2:**
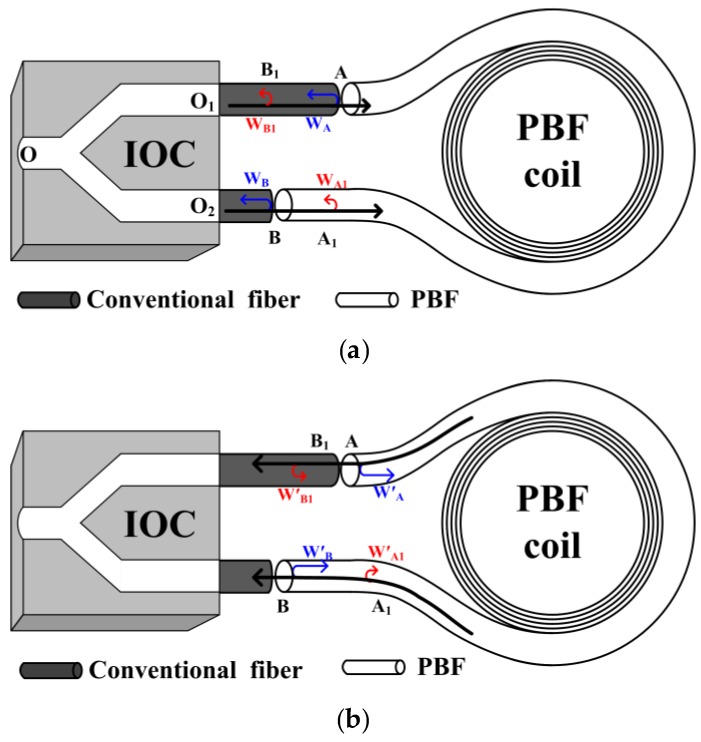
Secondary waves induced by back-reflection and backscatter of primary waves. (**a**) Before the primary waves enter the PBF coil; (**b**) After the primary waves have propagated through 1 loop of the PBF coil.

As illustrated in Figure 2a, before the primary-wave (black arrows) output from the IOC enters the PBF coil, secondary waves (W_A_ and W_B_) are generated owing to the strong back-reflection at fusion splicing points A and B. W_A_ has an optical path of *L*(W_A_) = *n*_IOC_|OO_1_| + *n*_SiO2_|O_1_A| from the common input/output end O. *n*_IOC_ and *n*_SiO2_ is, respectively, the refractive index of the IOC and conventional fiber. It is well known that the backscattering is randomly distributed along the fiber, and each backscattering point causes a secondary wave, but not all of those backscattering-induced secondary waves are able to interfere with W_A_, because it is the broad-spectrum light in the optical circuit and the interference intensity is maximum when the optical path difference (OPD) is 0, but decreases dramatically and rapidly as the OPD increases [14]. Therefore, if a secondary wave W_A1_ induced by the backscattering at point A_1_ has an optical path of *L*(W_A1_)= *n*_IOC_|OO_2_| + *n*_SiO2_|O_2_B| + *n*_air_|BA_1_|from the common input/output end O and *L*(W_A1_) = *L*(W_A_), then the interference occurs between W_A_ and W_A1_ and the intensity is maximum that is given by the first term in:

(1) $$\begin{array}{ll} I_{BSC} & =2I_{in}^{}\alpha_{in}^{}\alpha_{splicing}^{}{\left(R_{A}R_{A1}\right)}^{1/2}\cos(\Phi_{m}+\varphi_{1})+2I_{in}^{}\alpha_{in}^{}{\left(R_{B}R_{B1}\right)}^{1/2}\cos(\Phi_{m}+\varphi_{2})\\ & +2I_{in}^{}\alpha_{in}^{}\alpha_{splicing}^{2}\alpha_{coil}^{2}{\left(R_{A}R_{A1}\right)}^{1/2}\cos(\Phi_{m}+\varphi_{3})+2I_{in}^{}\alpha_{in}^{}\alpha_{splicing}^{3}\alpha_{coil}^{2}{\left(R_{B}R_{B1}\right)}^{1/2}\cos(\Phi_{m}+\varphi_{4}) \end{array}$$

which equation describes the summed intensity (as detected by the detector) of all the interference phenomena involved (that is, *I*_BSC_). Here *n*_air_ is the refractive index of the PBF. In Equation (1), *I*_in_ denotes the light intensity output from the IOC. Further, α_in_, α_splicing_, and α_coil_ represent the losses at the input channel (from the IOC pigtail to detector), fusion splicing at points A and B, and PBF coil, respectively. The typical values of α_in_, α_splicing_, and α_coil_ are ~8 dB, ~1.5 dB and ~6.6 dB for a 300-m PBF coil, respectively. Parameters *R*_A_ and *R*_B_ represent the back-reflection coefficients at points A and B, respectively. Similarly, *R*_A1_ and *R*_B1_ represent the backscattering coefficients at points A_1_ and B_1_, respectively. Parameters *φ*_1_, *φ*_2_, *φ*_3_, and *φ*_4_ denote the random phases between the two secondary waves involved in the interference. *Ф*_m_ is the modulation phase.

Interference between W_B_ and W_B1_ can similarly be examined, and the resulting interference intensity is given by the second term in Equation (1). On the other hand, as illustrated in Figure 2b, when the primary-wave output from the PBF coil after propagating through the coil once, backward secondary waves (W′_A_, W′_B_, W′_A1_, W′_B1_) arise, and interference can also occur between W′_A_ and W′_A1_, W′_B_ and W′_B1_ with the intensities given by the last two terms in Equation (1). In fact, there also exist other backward secondary waves that propagate over multiple loops; however, their contribution to the BSC error is very small, and the relevant terms have been neglected in Equation (1).

In the standard FOG operation, a square wave with eigenfrequency *f*_τ_ and amplitude of ±π/8, and a sawtooth wave with amplitude π are added and applied to the IOC to modulate primary waves for coherence detection and closing-loop operation [14]. The demodulation value of the interference intensity between the primary waves indicates the angle velocity of the FOG. The back-reflection-induced and backscatter-induced secondary waves also propagate through the IOC, and therefore, these waves are also modulated by the square and sawtooth waves; thus, the modulation phase *Ф*_m_ in Equation (1) includes two parts: the square-wave-induced phase (*Ф*_m_SQ_) and the sawtooth-wave-induced phase (*Ф*_m_SA_). Because the secondary waves propagate twice through the same branch of the IOC, *Ф*_m_SQ_ has an amplitude of ±π/2 and frequency of *f*_τ_, and *Ф*_m_SA_ has an amplitude of 4π.

According to the theory of coherence detection, because *Ф*_m_SQ_ has the same modulation frequency (*f*_τ_) as that of the primary waves, the demodulation value of *I*_BSC_ cannot be separated from the angle velocity of the FOG which is the demodulation result of the primary waves’ interference, thus leading to a bias error (namely, the BSC error) [14]. On the other hand, *Ф*_m_SA_ leads to BSC error’s variation with the sawtooth wave, and there are two complete periods when the sawtooth wave changes from 0 (rad) to π (rad). Under extreme conditions, the maximum BSC error is given by:

(2) $$\mathrm{BSC}{\mathrm{Error}}_{\max}=(\alpha_{splicing}^{}\sqrt{R_{A}R_{A1}}+\sqrt{R_{B}R_{B1}}+\alpha_{splicing}^{2}\alpha_{coil}^{2}\sqrt{R_{A}R_{A1}}+\alpha_{splicing}^{3}\alpha_{coil}^{2}\sqrt{R_{B}R_{B1}})/\left(2\pi LD/\lambda c\cdot \alpha_{splicing}^{2}\alpha_{coil}^{}\right)$$

where *D* and *L* represent the diameter and fiber length of the coil, respectively, *λ* the wavelength, and *c* the velocity of light.

## 3. Experimental Results

**Figure 3:**
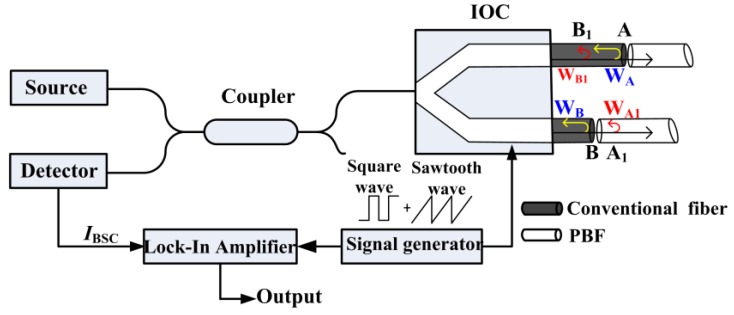
Experimental setup for measuring the BSC error.

To verify the existence of the BSC error and measure its magnitude in a practical FOG, we promoted a method and established the corresponding experimental setup, as illustrated in Figure 3, where the light launched from an amplified spontaneous emission (ASE) source enters the IOC through a single-mode fiber coupler, and it travels backward to the detector owing to the back-reflection and backscattering. The ASE source has a power of ~5 mW and the IOC is a proton-exchanged LiNbO_3_ circuit with the half-wave voltage of ~5 V. In fact, the setup mainly aims at determining the first two terms in Equations (1) or (2), which are the dominant BSC error terms. Moreover, our setup has a configuration that is nearly identical to the FOG configuration; square and sawtooth waves are applied to the IOC, and a lock-in amplifier is used to demodulate the interference intensity (*I*_BSC_) at the detector so as to better simulate the actual FOG operation. The one difference in our case is that two lengths of PBFs (and not a PBF coil) are connected to the two IOC pigtails to eliminate interference between the primary waves whose demodulation value cannot be separated from the measurement results. From the aspect of BSC error determined by the first two terms in Equation (1), this difference does not matter, because this part of the BSC error does not depend on the subsequent PBF coil. Considering the IOC’s half-wave voltage and the signal generator’s single-end output, we set the square wave frequency to 500 kHz with an amplitude of ±1.25 V, and the sawtooth wave is set to vary from 0 V to 10 V. Based on the analysis mentioned above, the demodulation result of *I*_BSC_ should have two sinusoidal periods when the sawtooth wave changes from 0 V to 10 V, and its peak-to-peak value can be easily resolved, which value directly reflects *I*_in_α_splicing_(*R*_A_*R*_A1_)^1/2^ or *I*_in_(*R*_B_*R*_B1_)^1/2^ or their sum according to which splicing point exists in the system.

**Figure 4:**
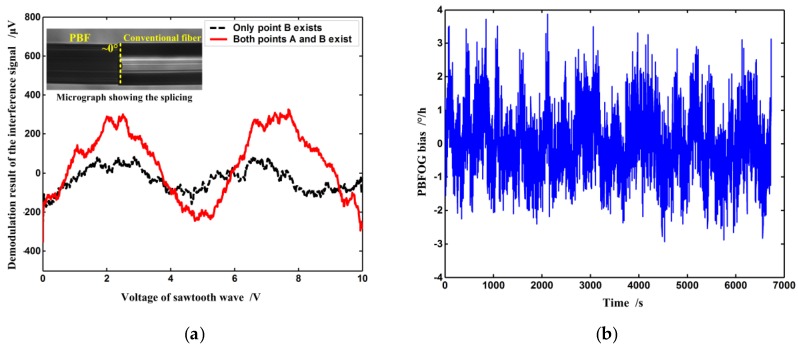
Test results of (**a**) the BSC error and (**b**) bias stability in the PBFOG when the PBFs are connected to the IOC pigtails through normal fusion splicing. Inset: micrograph showing the fusion splicing between PBF and conventional fiber at point A or B.

First, a length of PBF is normally connected to the IOC pigtail at point B through fusion splicing. The micrograph of the splicing between the PBF and IOC pigtail is shown in the inset in Figure 4a. Since it is 0° splicing, the secondary wave W_B_ is generated and its interference with W_B1_ occurs simultaneously. The lock-in amplifier’s output after demodulation of the interference intensity *I*_BSC_ is described by the dashed curve in Figure 4a where the fixed bias induced by such phenomena as Earth rotation has been taken out. Obviously, the output varies with the voltage of the sawtooth wave, and there are two periods when the voltage changes from 0 V to 10 V, a result that agrees well with the abovementioned analysis. The peak-to-peak value of the output is ~130 μV, which implies that (*R*_B_*R*_B1_)^1/2^ ~3.5 × 10^−^^7^, and therefore, the maximum BSC error in this case is ~1.36°/h according to Equation (2) for a 300-m PBF coil with a diameter of 10 cm. Next, a second PBF is also similarly connected to the other IOC pigtail at point A by fusion splicing. The resulting output is described by the solid line in Figure 4a. The peak-to-peak value of the output is ~450 μV, and consequently, the total maximum BSC error is ~4.7°/h according to Equation (2). The experimental results indicate that interference between W_A_ and W_A1_ contributes more to the BSC error than that between W_B_ and W_B1_, which can be explained by the fact that the backscatter coefficient in the PBF is higher than that in conventional fibers [15].

In a real closed-loop PBFOG, the BSC error can seriously affect the bias stability. Bias stability is defined as the random variation in bias as computed over specified finite sample time and averaging time intervals according to IEEE standard [16]. The secondary waves W_A_ and W_A1_, W_B_ and W_B__1_ are not reciprocal, because they propagate along different optical paths (see Figure 2a). The environment (temperature, vibration, and so on) definitely has different influences on different optical paths, as a result, their phase differences (*φ*_1_, *φ*_2_) are random environment-dependent variables. When those secondary waves interfere, their interference intensities and the induced BSC error would also vary randomly, so the PBFOG bias becomes unstable if it includes this error. In order to verify the BSC error’s influence on PBFOG bias stability, a real PBF coil, instead of the two lengths of PBFs, is connected to the IOC pigtails with normal fusion splicing on the basis of the experimental setup in Figure 3, and forms a complete PBFOG. A signal-processing electronic circuit substitutes the lock-in amplifier and signal generator to implement the digital closing-loop operation in the PBFOG [14]. The test result of the PBFOG bias is shown in Figure 4b, where the fixed bias induced by such as earth rotation has also been taken out. The test result indicates that the bias remarkably fluctuates and the corresponding bias stability is ~0.75°/h (standard deviation, 10 s integration time).

**Figure 5:**
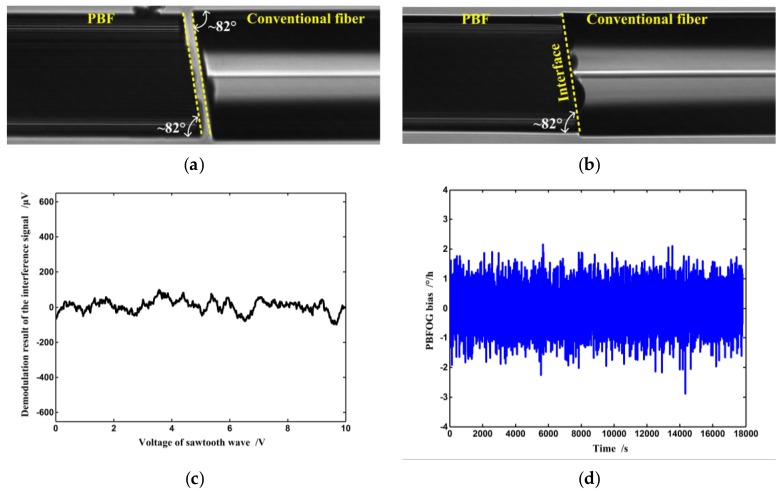
Micrographs showing (**a**) the cleaved angles of pigtails of the PBF coil and IOC and (**b**) cross section of the corresponding point after fusion splicing; (**c**) Test results of the BSC error and (**d**) bias stability in the PBFOG when the PBF coil is connected to the IOC pigtails through ~8° fusion splicing.

Although backscatter within the fiber cannot be eliminated, back-reflection can be reduced through an angled fiber end face in fiber termination in order to suppress the BSC error. A tilted angle of 8° is a commonly used value for conventional fiber and it is often applied in fiber connectors to suppress back-reflection [17]. Although a larger cleavage angle can further reduce the back reflection, this will come at the cost of increased splicing loss, so in the experimental FOG, both the pigtails of the IOC and PBF coil are cleaved at a tilted angle of ~8° to guarantee both the suppression of back-reflection at the interface and the proper fusion splicing loss, as shown in Figure 5a [7,9,10,11,18]. After fusion splicing, according to the test results, the reflectance at the interface (see Figure 5b) is reduced from greater than −20 dB to ~−54 dB, so the intensity of both W_A_ and W_B_ decreases to be negligible compared to the primary waves. Under this condition, the BSC error is also negligible according to the experimental result, as illustrated in Figure 5c that gives the test result of the BSC error; the corresponding PBFOG performance is shown in Figure 5d. Obviously, when the BSC error is suppressed, the bias stability is dramatically improved to ~0.4°/h (standard deviation, 10 s integration time). Therefore, we can conclude that the BSC error is an important bias error source that seriously affects the PBFOG performance, and it has to be addressed before any practical application of the PBFOG is considered.

## 4. Conclusions

In summary, we found and investigated a bias error (namely, the BSC error) in the PBFOG that is caused by interference between back-reflection-induced and backscatter-induced secondary waves. The BSC error was theoretically analyzed and experimentally verified. Our experimental results showed a BSC error of ~4.7°/h for a 300-m PBF coil with a diameter of 10 cm. Therefore, the BSC error is an important error source in the PBFOG, and it seriously affects the long-term stability of PBFOG. This error has to be addressed before the practical application of the PBFOG is considered, and our future work will focus on further studying possible measures to suppress the BSC error to improve the bias stability of the PBFOG.
